# Predictors of HIV infection: a prospective HIV screening study in a Ugandan refugee settlement

**DOI:** 10.1186/s12879-016-2021-1

**Published:** 2016-11-23

**Authors:** Kelli N. O’Laughlin, Dustin J. Rabideau, Julius Kasozi, Robert A. Parker, Nirma D. Bustamante, Zikama M. Faustin, Kelsy E. Greenwald, Rochelle P. Walensky, Ingrid V. Bassett

**Affiliations:** 1 Department of Emergency Medicine, Brigham and Women’s Hospital, 75 Francis Street, Boston, MA 02115 USA; 2 Medical Practice Evaluation Center, Department of Medicine, Massachusetts General Hospital, 50 Staniford Street, 9th Floor, Boston, MA 02114-2698 USA; 3 Harvard Medical School, 25 Shattuck Street, Boston, MA 02115 USA; 4 Harvard Humanitarian Initiative, Cambridge, MA USA; 5 MGH Biostatistics Center, Massachusetts General Hospital, Boston, MA USA; 6 Harvard University Center for AIDS Research (CFAR), Boston, MA USA; 7 United Nations High Commissioner for Refugees, Representation in Uganda, PO Box 3813, Kampala, Uganda; 8 Division of General Medicine, Massachusetts General Hospital, Boston, MA USA; 9 Department of Emergency Medicine, Massachusetts General Hospital, Boston, MA USA; 10 Bugema University, Kasese Campus, PO Box 6529, Kampala, Uganda; 11 Division of Infectious Disease, Massachusetts General Hospital, Boston, MA USA; 12 Division of Infectious Disease, Brigham & Women’s Hospital, Boston, MA USA

**Keywords:** HIV, Refugee, HIV testing, HIV screening, sub-Saharan Africa

## Abstract

**Background:**

The instability faced by refugees may place them at increased risk of exposure to HIV infection. Nakivale Refugee Settlement in southwestern Uganda hosts 68,000 refugees from 11 countries, many with high HIV prevalence. We implemented an HIV screening program in Nakivale and examined factors associated with new HIV diagnosis.

**Methods:**

From March 2013-November 2014, we offered free HIV screening to all clients in the Nakivale Health Center while they waited for their outpatient clinic visit. Clients included refugees and Ugandan nationals accessing services in the settlement. Prior to receiving the HIV test result, participants were surveyed to obtain demographic information including gender, marital status, travel time to reach clinic, refugee status, and history of prior HIV testing. We compared variables for HIV-infected and non-infected clients using Pearson’s chi-square test, and used multivariable binomial regression models to identify predictors of HIV infection.

**Results:**

During the HIV screening intervention period, 330 (4%) of 7766 individuals tested were identified as HIV-infected. Refugees were one quarter as likely as Ugandan nationals to be HIV-infected (aRR 0.27 [0.21, 0.34], *p* < 0.0001). Additionally, being female (aRR 1.43 [1.14, 1.80], *p* = 0.002) and traveling more than 1 h to the clinic (aRR 1.39 [1.11, 1.74], *p* = 0.003) increased the likelihood of being HIV-infected. Compared to individuals who were married or in a stable relationship, being divorced/separated/widowed increased the risk of being HIV-infected (aRR 2.41 [1.88, 3.08], *p* < 0.0001), while being single reduced the risk (aRR 0.60 [0.41, 0.86], *p* < 0.0001). Having been previously tested for HIV (aRR 0.59 [0.47, 0.74], *p* < 0.0001) also lowered the likelihood of being HIV-infected.

**Conclusions:**

In an HIV screening program in a refugee settlement in Uganda, Ugandan nationals are at higher risk of having HIV than refugees. The high HIV prevalence among clients seeking outpatient care, including Ugandan nationals and refugees, warrants enhanced HIV screening services in Nakivale and in the surrounding region. Findings from this research may be relevant for other refugee settlements in Sub-Saharan Africa hosting populations with similar demographics, including the 9 other refugee settlements in Uganda.

## Background

Sub-Saharan Africa is home to approximately 3 million refugees and asylum-seekers as well as 25.8 million adults living with HIV/AIDS [1, 2]. Despite the large number of refugees and the focused burden of HIV in sub-Saharan Africa, the prevalence of HIV among refugees is unknown. Furthermore, refugees face disrupted community social structures and their ability to cope with difficult living conditions is compromised [3, 4]. These factors may increase their vulnerability for exposure to HIV and result in hardships when attempting to access HIV-related services [4, 5]. Targeted interventions to diagnose HIV-infected refugees and engage them in medical care will likely improve survival and reduce transmission of disease. These data will be essential to initiate HIV treatment for all who need it as we aim to end the global AIDS epidemic [6].

Working with local collaborators from Medical Teams International (MTI) and the United Nations High Commissioner for Refugees (UNHCR), we established a refugee-centered research team focused on improving delivery of HIV services for refugees and nationals in Nakivale Refugee Settlement in southwestern Uganda. We conducted a clinic-based HIV screening intervention study at Nakivale Health Center [7]. Our primary objective for this analysis was to evaluate the correlates of HIV infection of those tested during this screening intervention.

## Methods

### Study design

This analysis is part of a larger study that compared a Standard of Care period (January 15, 2013 to March 13, 2013) to an Intervention period (March 14, 2013 to November 15, 2013) to assess the impact of an HIV screening intervention at Nakivale Health Center [7]. The single intervention was that clients, including refugees and Ugandan nationals, were offered free HIV testing by dedicated research staff while waiting for their outpatient department clinic visit. Approximately 21% of all-comers to the outpatient department participated in HIV screening [7]. Data was not collected on those who did not participate in HIV screening. After signing written consent forms, participants were orally administered a structured questionnaire by research assistants. Data was entered directly into a secure laptop computer. Survey data included information such as gender, marital status, travel time to reach clinic, refugee status, and history of prior HIV testing. After the survey was complete, participants were counseled regarding their HIV test result and were given information about HIV transmission with specific instructions on HIV prevention and on how to follow-up with HIV clinic as applicable. For purposes of this research, we used data from the ongoing intervention, which includes clients who tested from March 14, 2013 through November 14, 2014, to evaluate demographic and socioeconomic correlates of being diagnosed with a new HIV infection during the screening intervention.

### Study setting

This study was conducted in Nakivale Refugee Settlement in southwestern Uganda. Nakivale was established in 1960 to accommodate Rwandan refugees. It spans 71 square miles and hosts 68,000 refugees from 11 countries [8]. Most refugees are from four countries: the Democratic Republic of the Congo (52%), Somalia (16%), Burundi (15%) and Rwanda (15%) [8]. Nakivale Health Center is the largest health clinic in the settlement and is operated by the non-profit organization Medical Teams International. Services at Nakivale Health Center are accessed by refugees living in the settlement, and importantly, also by Ugandan nationals living in and around the settlement. There is an outpatient department that is open on weekdays where clinicians care for approximately 80 adult patients per day.

At Nakivale Health Center, HIV testing is free to all clients and is performed using serial rapid HIV tests as specified by the Uganda HIV Rapid Test Algorithm [9]. Antiretroviral therapy (ART) is provided free of charge (during the time of this research ART was initiated at CD4 ≤ 350/mm3 or WHO Stage III/IV based on 2010 WHO guidelines [10]). Pre-ART clients are monitored with monthly clinic visits and are given co-trimoxazole prophylaxis [11]. Three smaller clinics in the settlement offer free HIV screening, pre-ART clinics, and ART clinics. While HIV prevalence in Nakivale is unknown, we found a prevalence of 4.5% during our initial HIV screening intervention study conducted at Nakivale Health Center [7]. The prevalence of HIV from the refugees’ countries of origin ranges from 0.5 to 5.9% [12], and the prevalence in the surrounding region of Uganda is 7.3% [13].

### Subject selection

Study participants were recruited by research assistants through the use of HIV-related educational presentations conducted twice each morning to clients in the outpatient department waiting area at Nakivale Health Center. Eligibility criteria included: 1) 18 years or older; 2) ability to provide informed consent in one of four languages: Swahili, Kinyarwanda, Runyankore, or English; 3) not already known to be HIV-infected; and 4) no prior participation in this research study. Clients were invited to obtain a free rapid HIV test and were given the test result while waiting for their outpatient department clinic visit. Prior to the HIV screening intervention, the standard of care was for clients to request a free HIV test or to be referred for diagnostic HIV testing based on clinical suspicion. In these instances, the HIV test was conducted in the outpatient department laboratory with results provided to clients later in the day or the following morning.

### Data collected/classification of endpoints

Four research assistants hired for the study performed study recruitment, consent, surveying and rapid HIV testing. These steps were offered in the four languages listed above. Research assistants had experience in HIV counseling and were trained in rapid HIV testing by members of the Center for Disease Control and Prevention (CDC) in Uganda. Participants were tested in a semi-permanent two-room tent approximately 25 m from the outpatient department. While the HIV test was conducted, and prior to return of HIV test results, research assistants performed the survey to collect demographic and socioeconomic information. After the survey was complete, prior to leaving the HIV testing area, research assistants gave study participants their HIV test result. Participants were counseled regarding their diagnosis and given appropriate instructions on how to enroll in HIV clinical care when indicated.

### Statistical methods

We report descriptive data as frequencies or medians with interquartile range, as appropriate. For comparisons between groups (HIV-infected and non-infected) for univariate analyses of variables, we used Pearson’s chi-square test. We then fit multivariable binomial regression models using a log link function to estimate unadjusted and adjusted relative risk of testing as HIV-infected. Variables were considered for inclusion in the multivariate model if univariate screening *p*-value was less than 0.10 and were ultimately included in the model if the adjusted *p*-value was less than 0.05. Due to the inherent association between the variables “refugee status”, “live in Nakivale”, and “country of origin”, we considered only “refugee status” for inclusion into our multivariable model. Exact 95% confidence intervals (CI) for proportions were calculated using the Pearson-Klopper method. Statistical analyses were performed using SAS 9.4 (Cary, NC, USA) and R version 3.1.2 (www.r-project.org).

## Results

During the study period, we have records of 23,016 encounters at the clinic (which may include multiple encounters of a single individual). Of these encounters, 65% were female, the median age was 32 (IQR: 25–43), and 92% were refugees. Though study recruitment occurred in the outpatient department clinic waiting area, not all clients screened for HIV attended the outpatient department clinic. Definitive HIV testing results were obtained for 7766 subjects of which 52% were female, median age was 28 years (IQR: 22–37), and 69% were refugees (Table 1). Of all clients tested, 85% reported that they live in Nakivale, and the median number of years living in Nakivale for this group was 4 (IQR: 1–8). The majority of enrollees were married or living with someone (65%) and had some primary school or no formal schooling (64%). Approximately three-quarters (73%) reported prior HIV testing. One third of clients traveled more than one hour to Nakivale clinic.

**Table 1:** Baseline characteristics of cohort

Variable	Overall (*N* = 7766) % (*n*/*N*)	HIV-infected (*N* = 330) % (*n*/*N*)	Non-HIV-infected (*N* = 7436) % (*n*/*N*)	*P*-value*
Female	52% (4016/7766)	63% (208/330)	51% (3808/7436)	<0.0001
Age category				0.10
< 30	53% (4132/7766)	49% (160/330)	53% (3972/7436)	
30 to <40	26% (2020/7766)	29% (96/330)	26% (1924/7436)	
40 to <50	13% (1029/7766)	16% (54/330)	13% (975/7436)	
≥ 50	8% (585/7766)	6% (20/330)	8% (565/7436)	
Refugee	69% (5318/7743)	34% (113/329)	70% (5205/7414)	<0.0001
Live in Nakivale	85% (6430/7534)	63% (205/323)	86% (6225/7211)	<0.0001
Country of origin				<0.0001
Uganda	32% (2457/7763)	66% (218/330)	30% (2239/7433)	
Rwanda	31% (2395/7763)	17% (56/330)	31% (2339/7433)	
DRC	20% (1580/7763)	9% (30/330)	21% (1550/7433)	
Burundi	13% (987/7763)	4% (14/330)	13% (973/7433)	
Other†	4% (344/7763)	4% (12/330)	5% (332/7433)	
Relationship status				<0.0001
Married/living together	65% (4872/7534)	64% (207/323)	65% (4665/7211)	
Single	24% (1832/7534)	11% (37/323)	25% (1795/7211)	
Divorced/separated/widowed	11% (830/7534)	25% (79/323)	10% (751/7211)	
Education				0.19
No school	20% (1524/7534)	20% (64/323)	20% (1460/7211)	
Some primary school	44% (3267/7534)	48% (156/323)	43% (3111/7211)	
Completed primary school	16% (1224/7534)	16% (51/323)	16% (1173/7211)	
Higher than primary school‡	20% (1519/7534)	16% (52/323)	21% (1467/7211)	
HIV knowledge (all correct)§	34% (2544/7542)	38% (122/323)	34% (2422/7219)	0.12
Previous HIV test	73% (5538/7536)	65% (210/322)	74% (5328/7214)	0.0006
More than 1 h to clinic	33% (2497/7548)	53% (171/323)	32% (2326/7225)	<0.0001

Note: Denominators vary due to participant non-response

*Abbreviations*: *DRC* Democratic Republic of the Congo

* *P*-value based on Pearson chi-square test

† Other includes Somalia, Eritrea, Ethiopia, Sudan, Kenya, Tanzania, Senegal, and Zaire

‡ Includes some/completed secondary school, vocational school, certificate program, bachelors, and post graduate

§ Questionnaire included 4 questions [correct answer]: (1) Do you think that a healthy-looking person can be infected with HIV, the virus that causes AIDS? [Yes]; (2) Can a person get HIV by sharing a meal with someone who is infected? [No]; (3) Can a pregnant woman infected with HIV or AIDS transmit the virus to her unborn child? [Yes]; (4) Can a woman with HIV or AIDS transmit the virus to her newborn child through breastfeeding? [Yes]

A total of 330 subjects (4%) were found to be HIV-infected, of whom 63% were female, the median age was 30 (IQR: 24–38), and 34% were refugees. The HIV-infected cohort had a higher proportion of participants who were female, national (i.e. Ugandan non-refugees), divorced/separated/widowed, and who traveled more than 1 h to the Nakivale clinic; it had a lower proportion of subjects who were refugees, residents of Nakivale, single, and previously tested for HIV (Table 1). Country of origin was significantly different between HIV-infected and non-HIV-infected subjects (*p* < 0.0001). Most of this association was driven by the higher proportion of HIV-infected Ugandans. This was verified by excluding Ugandans from the analysis of country of origin by HIV test result using a Pearson chi-square test (*p* = 0.09). Ugandans who reported living in the settlement were less likely to be HIV-infected compared to Ugandans living outside the settlement (7 vs. 11%, *p* = 0.009). Of the three predominant countries of origin represented by the refugees that participated in HIV testing, the HIV prevalence among Rwandans was 2.3% (56/2395; 95% CI: 1.7–3.1%), among Congolese was 1.9% (30/1580; 95% CI: 1.2–2.7%), and among Burundians was 1.4% (14/987; 95% CI: 0.7–2.4%). The 12 remaining HIV-infected refugees were too few to adequately assess HIV prevalence by country group.

According to the final multivariable model, refugees were approximately one quarter as likely as Ugandan nationals to be HIV-infected (aRR 0.27, CI 0.21–0.34). Additionally, being female (aRR 1.43, CI 1.14–1.80), divorced/separated/widowed (aRR 2.41, CI 1.88–3.08), and traveling more than 1 h to the clinic (aRR 1.39, CI 1.11–1.74) increased the likelihood of being HIV-infected, while being single (aRR 0.60, CI 0.41–0.86) and having been tested previously for HIV (aRR 0.59, CI 0.47–0.74) lowered the likelihood of being HIV-infected (all *p*-values < 0.05, Table 2). Age, education, and HIV knowledge did not meet the univariate screening criteria of *p* < 0.10.

**Table 2:** Predictors of HIV infection

Variable	Unadjusted		Adjusted	
	Relative Risk [95% CI]	*P*-value	Relative Risk [95% CI]	*P*-value
Refugee	0.24 [0.19, 0.30]	<0.0001	0.27 [0.21, 0.34]	<0.0001
Female	1.59 [1.27, 1.99]	<0.0001	1.43 [1.14, 1.80]	0.002
Relationship status		<0.0001		<0.0001
Married/living together	REF		REF	
Single	0.48 [0.33, 0.68]		0.60 [0.41, 0.86]	
Divorced/separated/widowed	2.24 [1.74, 2.88]		2.41 [1.88, 3.08]	
Previous HIV test	0.68 [0.54, 0.85]	0.0006	0.59 [0.47, 0.74]	<0.0001
More than 1 h to clinic	2.28 [1.83, 2.82]	<0.0001	1.39 [1.11, 1.74]	0.003

Note: Results are based on binomial regression

## Discussion

This study of routine HIV screening at Nakivale Health Center identified participant characteristics that were associated with likelihood of HIV infection. Clients had an increased likelihood of being HIV-infected if they were female, divorced/separated/widowed, or traveled more than one hour to clinic. Conversely, clients had a decreased likelihood of being HIV-infected if they were a refugee, single, or if they had previously tested for HIV. Refugees were one quarter as likely to be HIV-infected compared to Ugandan nationals.

Refugee settlements often have lower HIV prevalence than surrounding host communities [14]. This finding is reflected in our data – refugees have a much lower prevalence than Ugandan nationals (2 vs. 9% respectively). This may be a sign of the lower HIV prevalence in the refugees’ countries of origin compared to Uganda. It could be that refugees in Nakivale have fewer sexual partners overall and fewer casual sexual partners than Ugandan nationals, as was the case in a series of cross-sectional HIV behavioral surveillance surveys in Kenya and Uganda [15]. Ugandans who access HIV screening services in the refugee settlement may also be a particularly vulnerable subset of the national population. While conflict and forced displacement have not been shown to increase HIV prevalence [14, 16, 17], we observed that some sub-groups of refugees have increased prevalence compared to the HIV prevalence in their country of origin. Comparing the predominant sub-groups of refugees that tested for HIV in Nakivale to adult populations (ages 15–49) in their country of origin, we observed that Congolese refugees had a higher prevalence than adults in the Democratic Republic of the Congo (1.9 vs. 0.8%) and Burundian refugees had a higher prevalence than adults in Burundi (1.4 vs. 1.0%) [11]. Conversely, Rwandans refugees had a lower prevalence than adults in Rwanda (2.3 vs. 2.9%) [11]. While both the Burundian and Rwandan differences are consistent with the rates observed in our study (differences not statistically significant; both *p* > 0.05), the differences in prevalence for refugees from the Democratic Republic of the Congo should be further evaluated to better understand the characteristics and potential differences of the people accessing HIV testing. It is evident that HIV infection is endemic among both refugees and Ugandan nationals and that both groups would benefit from scaled-up HIV screening and clinical services in Nakivale.

Perhaps most notable in our data is the high prevalence of HIV among Ugandan nationals compared to refugees. Though Ugandan nationals do not have land ownership rights permitting them to live within Nakivale, many do (Table 1, 5318 report they are refugees, yet 6430 report they live in Nakivale) and others freely come and go from the settlement for job-related or personal reasons [18]. Our data reflect that HIV-infected Ugandans are more likely to report that they live outside of Nakivale Refugee Settlement. There is concern that some Ugandans may travel to Nakivale for commercial sex. If Ugandans with higher prevalence of HIV participate in commercial sex within Nakivale, this puts those living in the settlement at higher risk for HIV transmission. Integrated HIV programs could be an efficient and effective means to reach both of these groups in this setting [18].

We found a significantly higher likelihood of HIV among females participating in HIV screening compared to males. This is similar to findings reported in the 2011 Uganda AIDS Indicator Survey that showed HIV prevalence in Uganda to be higher among women than men (8.3 vs. 6.1% respectively) [13]. It is the case that young women are disproportionately affected by HIV in sub-Saharan Africa with women acquiring HIV 5 to 7 years earlier than men [19]. This could be more pronounced in refugee settlements as many women were displaced by armed conflict, and gender-based violence is known to be used as a weapon of war in this region [20]. A cross-sectional survey conducted in Northern Uganda in 2010 among transit camp residents showed higher likelihood of HIV infection for those with a non-consensual sexual debut [21]. Additionally, plural marriages in the refugee settlement could differentially impact the HIV prevalence of women.

Those who traveled more than 1 h to reach the clinic had a higher likelihood of HIV infection. Perhaps those who traveled further reflect a higher risk or sicker population who delayed testing until they were ill [22]. It has been demonstrated in sub-Saharan Africa that distance to clinic is a barrier to HIV service utilization [23, 24]. Assuming time to clinic is a proxy for distance to clinic, it may be that those living farther from clinic have received less HIV preventative counseling and less treatment for ailments such as sexually transmitted infections and therefore have a higher risk of being infected.

These data must be interpreted within the context of the study design. The study was conducted in one refugee settlement. Though there are likely to be similarities in other refugee settlements, particularly those in Uganda, it is probable that differences exist between correlates of HIV infection among populations with different demographics and countries of origin. Information about study participants was collected using survey methods, and as such the data were not verifiable. There was a potential sampling bias of those living closer to clinic or those willing and able to travel to clinic to access HIV screening services. Another sampling bias may be that those accessing testing could be more likely to be sick with an HIV-related illness as they were physically present at the health center; this would lead to an overestimate of HIV prevalence. This potential bias is particularly important when we compare HIV prevalence of sub-groups by country; the differences demonstrated in this study could be explained by this bias. Though free HIV screening was offered to all clients waiting for an outpatient department clinic visit, less than a quarter of clients presenting for an outpatient department clinic visit opted to screen for HIV. Reasons for declining to participate in HIV testing may have included fear of finding out their HIV status, fear of stigma, or having a recent HIV test and/or known HIV status. It may have been that Ugandan nationals who opted to test for HIV in Nakivale were at higher or lower risk of HIV infection than Ugandans testing elsewhere. Despite these limitations, these data are informative and could help to guide HIV screening and outreach services for this setting.

## Conclusion

This is the first study to assess correlates of HIV infection among clients accessing HIV screening in a refugee settlement. By understanding more about high-risk populations accessing HIV screening in Nakivale Refugee Settlement, we can better target future HIV screening interventions for this setting. Despite lower prevalence among refugees compared to Ugandan nationals, both groups have a high HIV prevalence, supporting the need for enhanced HIV screening services in Nakivale. Using an evidence-based approach to enhance HIV screening and treatment interventions among refugees will help ensure effective and efficient use of resources for this vulnerable population. While refugee settlements differ from location to location, lessons learned in this study may be relevant for other refugee settlements in Sub-Saharan Africa hosting populations with similar demographics including the 9 other refugee settlements in Uganda.
